# Excess mortality up to 7 years after low-trauma hip fracture in the largest urban region in Romania

**DOI:** 10.1007/s11657-026-01736-3

**Published:** 2026-07-31

**Authors:** Ramona Dobre, Cristina Capatina, Iulia Florentina Burcea, Catalin Cirstoiu, Cosmin Baciu, Catalina Poiana

**Affiliations:** 1 Carol Davila University of Medicine and Pharmacy, Bucharest, Romania; 2 C.I. Parhon National Institute of Endorinology, Bucharest, Romania; 3 Orthopedic Department, Bucharest University Emergency Hospital, Bucharest, Romania; 4 ICU Department Floreasca Emergency Clinical Hospital, Bucharest, Romania

**Keywords:** Hip fracture, Long-term mortality, Survival

## Abstract

***Summary*:**

Hip fracture is the most severe complication of osteoporosis. We followed up patients with hip fracture for up to 7 years. Compared with the general population, the overall mortality rate ratio was approximately 9.27 over the follow-up period. Our findings justify the need for urgent improvement of osteoporosis and hip-fracture care.

**Introduction:**

Hip fracture is the most severe complication of osteoporosis, being associated with substantial morbidity, loss of independence, and increased long-term mortality. Data on excess mortality after hip fracture in the population from Eastern Europe is very limited. The objective of the study is to quantify excess mortality after hip fracture by comparing observed with expected mortality derived from age-specific population data for the largest urban region in Romania.

**Methods:**

We performed a longitudinal observational cohort study including patients hospitalized with hip fracture followed longitudinally up to 7 years after the fracture.

**Results:**

A total of 1977 hip fracture patients (1452 women) aged ≥ 40 years were included. Between 2018 and 2024, 1460 deaths occurred (overall mortality 73.85); the overall crude mortality rate in the cohort was 215.6 per 1000 person-years. Compared with the general population, the overall age-specific mortality rate ratio (RR) was approximately 9.27 over the follow-up period. Cohort crude mortality rates were highest in the first post-fracture year and then decreased but remained elevated all across follow-up with overall annual cohort-to-population RRs of 5.74 to 18.10. In univariate Cox regression analyses, female sex was associated with a lower risk of mortality (HR 0.84, 95% CI 0.75–0.94, *p* = 0.002), while conservative management was associated with a markedly increased risk of death compared with surgical treatment (HR 1.95, 95% CI 1.72–2.21, *p* < 0.001). In multivariate Cox proportional hazards analysis, conservative management remained independently associated with an increased risk of all-cause mortality (HR 2.01, 95% CI 1.77–2.28, *p* < 0.001), female sex remains independently associated with improved survival (HR 0.67, 95% CI 0.60–0.76, *p* < 0.001), and also increasing age is a strong independent predictor of mortality (HR 1.33, 95% CI 1.28–1.37, *p* < 0.001).

**Conclusions:**

Hip fracture is associated with a high and lasting mortality burden in Romanian patients. These findings justify the need for urgent improvement of osteoporosis care and hip fracture systems, aiming to improve survival and quality of life for this patient population.

**Supplementary Information:**

The online version contains supplementary material available at 10.1007/s11657-026-01736-3.

## Introduction

Hip fracture is probably the most severe complication of osteoporosis, being associated with substantial morbidity, loss of independence, and increased long-term mortality [1]. The characteristic for this type of fracture is that mortality after the event continues to be markedly increased compared with the general population despite improvements in surgical techniques, perioperative management, and rehabilitation pathways [2]. This excess mortality associated with hip fracture is a key outcome in contemporary hip fracture research [3].

Population-based studies and national registry analyses have confirmed that patients sustaining a hip fracture experience significantly higher mortality than expected for their age and sex with standardized mortality ratios (SMRs) higher in the first year after fracture but also an excess mortality that persists for many years thereafter [2]. Recent data suggest that this excess risk does not normalize even after 5 to 10 years, or longer follow-up, indicating a sustained survival disadvantage rather than a transient postoperative effect [4].

Men show substantially higher mortality than women, both in absolute terms and relative to the general population, despite lower fracture incidence [5]. Extracapsular fractures and conservative (non-surgical) management are generally linked to poorer survival, while timely surgical intervention and structured orthogeriatric care appear to mitigate, but not eliminate, excess mortality [1, 6, 7].

Most recent evidence on excess mortality after hip fracture originates from Western and Northern Europe and North America [2, 8–10]; in contrast, data from Eastern Europe remain sparse [2]. This lack of evidence is particularly relevant given regional differences in healthcare organization, access to surgical treatment, rehabilitation services, and secondary fracture prevention, all of which may influence survival outcomes [2].

Currently available nationwide data in Romania are limited to in-hospital mortality, as the existing reporting system does not support longitudinal follow-up after discharge [11], data on long-term mortality after hip fracture being extremely limited. To date, only one longitudinal study has evaluated post-fracture mortality up to 12-month follow-up period using a hospital-based cohort and demonstrated high early mortality with persistent long-term survival impairment and several characteristics like a very high rate of conservative treated fractures [5]. However, excess mortality relative to the general population has not previously been quantified, and age-specific comparisons with population mortality have not been reported.

The present study builds upon this previously described cohort [5] and aims to quantify excess mortality after hip fracture by comparing observed mortality with expected mortality derived from age-specific population data for the largest urban region in Romania. Given the persistent excess mortality reported after hip fracture in other European populations and the high fracture risk estimated for Romania by FRAX, the lack of long-term national data represents a major gap. The present study aims to address this limitation by quantifying excess mortality beyond 1 year in a Romanian cohort with complete longitudinal follow-up.

## Methods

### Study design and population

This is a longitudinal observational cohort study designed to evaluate long-term excess mortality following hip fracture by comparison with mortality in the general population. The study was conducted in the Bucharest–Ilfov region, the largest urban region in Romania, and included patients hospitalized with hip fracture that were followed-up longitudinally for up to 7 years after the index fracture.

The present analysis builds upon a previously described cohort [5], using identical inclusion criteria, data sources, and follow-up procedures, respectively, all patients aged 40 years and older admitted with a diagnosis of hip fracture, including intracapsular and extracapsular fractures, identified using ICD-10 codes S72.0, S72.1, and S72.2 in the Orthopedic Departments of 11 public hospitals (98.46% admission rate of the Romanian patients for hip fracture in the area of interest), between September 1, 2017, and August 31, 2018. Pathological fractures, high-energy trauma-related fractures, periprosthetic fractures, and re-admissions were excluded. Only the first hip fracture admission per patient was included in the analysis. Baseline demographic and clinical data were collected at the time of hospitalization and included age, sex, fracture type, and treatment strategy (surgical or non-surgical). The study received the approval of the hospitals’ Ethics Committees.

The primary outcome was all-cause mortality. For each patient, follow-up started at the date of index hospitalization for hip fracture and continued for up to 7 years. Event time was defined as the number of days from index hospitalization to death. Patients were administratively censored at 2555 days (7 years) or earlier at the date of death, whichever occurred first. Mortality data were obtained from the Directorate for Persons Record and Databases Management, main aggregator for populational data in our country (http://depabd.mai.gov.ro). The cause of death was not available.

The study included patients aged 40 years and older, consistent with the definition used in the parent population-based hip fracture cohort from which Romanian incidence estimates and subsequent FRAX-related epidemiological inputs were derived [12]. Although patients younger than 65 years were retained for methodological consistency, they represented only a small proportion of the cohort, and the mortality burden was predominantly concentrated in the older age groups. As a sensitivity analysis, we repeated the main mortality analyses in patients aged ≥ 65 years, including cumulative crude mortality and standardized mortality ratios by year of follow-up. This age threshold was selected to facilitate comparison with studies focusing on older hip fracture populations.

### Reference population

Age- and sex-specific mortality data for the general population were obtained from the National Institute of Statistics of Romania as aggregated tabulated data for the Bucharest–Ilfov region. Reference population data were available by calendar year, sex, and 5-year age group. Both resident population counts and number of deaths were stratified according to the same variables.

Annual mortality rates were calculated for each calendar year within each sex and 5-year age group stratum. Expected deaths were calculated by applying the corresponding calendar year-, sex-, and 5-year age group-specific mortality rates from the Bucharest–Ilfov reference population to the person-time accumulated by the hip fracture cohort in the matching strata. Thus, mortality rates were calculated within 5-year age groups, without finer age adjustment. No individual-level linkage with the reference population was performed.

### Excess mortality analysis

Excess mortality was quantified using standardized mortality ratios (SMRs), defined as the ratio of observed to expected deaths. Expected deaths were calculated by applying age-, sex-, and calendar year-specific mortality rates from the Bucharest–Ilfov reference population to the corresponding person-years at risk accumulated by the cohort. Therefore, the resulting SMRs represent indirectly standardized estimates accounting for differences in age and sex between the cohort and the general population. SMR analysis was considered the primary method for assessing excess mortality. Ninety-five percent confidence intervals for SMRs were estimated assuming a Poisson distribution of observed deaths. SMRs were calculated overall and stratified by time since fracture to evaluate changes in excess mortality over the 7-year follow-up period.

### Age-specific mortality rate ratios

Values are presented as number (percentage) unless otherwise stated. Age is expressed as mean ± standard deviation (SD). Age-specific mortality rates for the cohort were calculated as the number of deaths per 1000 person-years within each age group. Corresponding population mortality rates were calculated as deaths per 1000 persons within the same age group and calendar year. Age-specific mortality rate ratios were derived by dividing cohort mortality rates by population mortality rates within each age stratum. These estimates were used as descriptive stratum-specific comparisons and should not be interpreted as age-adjusted summary measures.

### Survival analysis

Survival probabilities were estimated using the Kaplan–Meier method for the overall cohort and stratified by sex, fracture type, and treatment management. Kaplan–Meier curves were used as unadjusted descriptive analyses of survival patterns. Age-stratified survival curves are presented as supplementary material.

Statistical analysis was performed with IBM SPSS Statistics, version 25 (IBM Corp., Armonk, NY, USA).

## Results

A total of 1977 hip fracture patients aged ≥ 40 years (women 73.44%, *n* = 1452) were included. Baseline characteristics of the cohort are shown in Table 1. During up to 7 years of individual follow-up after index hospitalization, 1460 deaths occurred, corresponding to 6772 person-years of observed follow-up. The overall crude mortality rate in the cohort was 215.6 per 1000 person-years**.** When compared with the Bucharest–Ilfov general population, the aggregated population mortality rate across the same age-band distribution (40–44 to ≥ 85 years) and years was 23.25 per 1000 persons**,** yielding an overall age-specific mortality rate ratio (RR) of approximately 9.27 over the follow-up period. Most patients were elderly, with the cohort predominantly composed of patients aged 70 years and older. Patients younger than 65 years represented only a small minority of the study population. This indicates a markedly higher mortality rate among hip fracture patients than among age-matched residents in the same region.

**Table 1:** Baseline characteristics of the hip fracture cohort

Characteristic	*n*	%
Total patients	1977	100
Age, years (mean ± SD)		79.2 ± 10.4
Age groups, years		
40–44	6	0.3
45–49	21	1.1
50–54	26	1.3
55–59	46	2.3
60–64	113	5.7
65–69	143	7.2
70–74	177	9.0
75–79	320	16.2
80–84	414	20.9
≥ 85	711	36.0
Sex		
F	1452	73.4
M	525	26.6
Fracture type		
Extracapsular	1179	59.6
Intracapsular	798	40.4
Management		
Conservative	377	19.1
Surgical	1600	80.9

*SD*, standard deviation

During up to 7 years of individual follow-up after index hospitalization, 1460 deaths occurred, corresponding to 6772 person-years of observed follow-up. Cumulative observed mortality increased progressively during follow-up. Overall cumulative mortality was 33.8% at 1 year, 51.0% at 3 years, 65.1% at 5 years, and 73.8% at 7 years. As expected, cumulative mortality increased with age, with the highest absolute mortality observed in the oldest age groups. These cumulative mortality estimates are presented as descriptive measures of absolute mortality burden, which are shown in Table 2, at fixed follow-up time points. The standardized comparison with the general population is provided by the SMR analysis.

**Table 2:** Cumulative observed mortality after hip fracture by age group and follow-up time

Age group, years	Baseline *N*	Deaths at 1 year, *n* (%)	Deaths at 3 years, *n* (%)	Deaths at 5 years, *n* (%)	Deaths at 7 years, *n* (%)
40–44	6	1 (16.7)	2 (33.3)	3 (50.0)	3 (50.0)
45–49	21	1 (4.8)	3 (14.3)	5 (23.8)	5 (23.8)
50–54	26	5 (19.2)	7 (26.9)	10 (38.5)	12 (46.2)
55–59	46	3 (6.5)	7 (15.2)	14 (30.4)	17 (37.0)
60–64	113	19 (16.8)	30 (26.5)	44 (38.9)	49 (43.4)
65–69	143	33 (23.1)	56 (39.2)	70 (49.0)	76 (53.1)
70–74	177	45 (25.4)	66 (37.3)	99 (55.9)	111 (62.7)
75–79	320	80 (25.0)	138 (43.1)	180 (56.3)	210 (65.6)
80–84	414	140 (33.8)	214 (51.7)	271 (65.5)	333 (80.4)
≥ 85	711	342 (48.1)	485 (68.2)	592 (83.3)	644 (90.6)
**Overall**	**1977**	**669 (33.8)**	**1008 (51.0)**	**1288 (65.1)**	**1460 (73.8)**

**Fig. 1:**
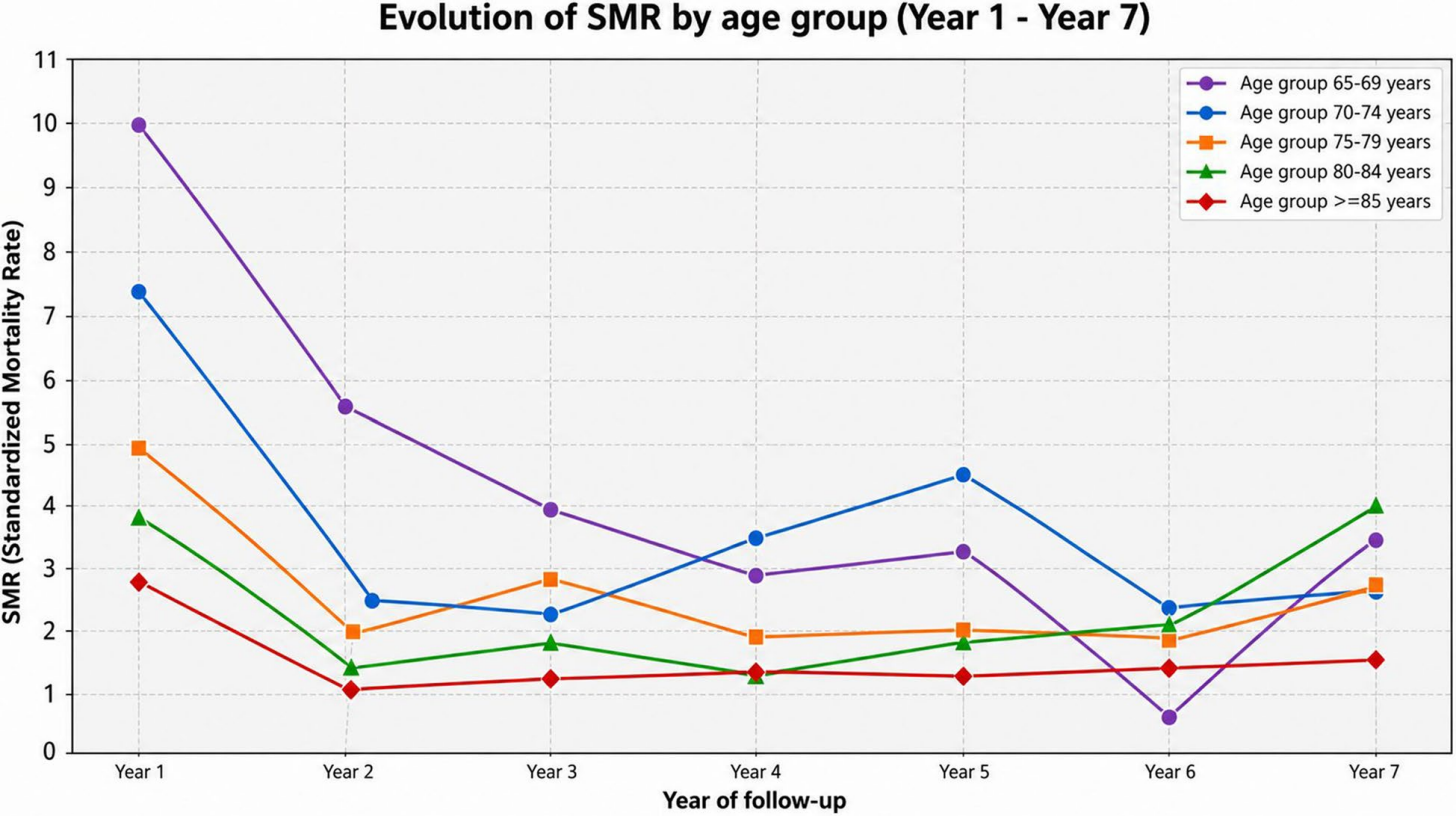
Standardized mortality ratios (SMR) by years since hip fracture. The figure illustrates the marked excess mortality in the first year after fracture, followed by a persistent but attenuated elevation over the subsequent follow-up period. SMR estimates in younger age groups, particularly below 70 years, should be interpreted cautiously because of small numbers of observed deaths and low expected mortality in the reference population

Standardized mortality ratios (SMRs) by years since fracture are shown in Table 3. A graphical representation of SMR by time since fracture is shown in Fig. 1. The data showed the expected pattern of marked early excess mortality with attenuation over time but with persistent elevation beyond the first year for the whole follow-up period. This SMR pattern aligns with the age-specific rate ratio findings: excess mortality is most pronounced early, immediately after the index fracture, but remains present across the full 7-year horizon. Cumulative age-specific SMR over the entire 0–7-year follow-up is shown in Table 4. Age-specific SMRs by follow-up interval are shown in Supplementary Table 1. SMR estimates in patients younger than 70 years should be interpreted with caution because of small numbers of observed deaths in several follow-up intervals and very low expected mortality in the reference population. These estimates are presented for completeness but should not be considered the main driver of the study conclusions, which are primarily supported by the overall SMR estimates and by the older age groups, where most fractures and deaths occurred.

**Table 3:** Standardized mortality ratios by time since hip fracture

Years since event	Observed deaths	Expected deaths	SMR	95% CI for SMR
First year	669	188.99	3.54	3.28–3.82
2^sd^	161	107.83	1.49	1.27–1.74
3rd	178	103.19	1.72	1.48–2.00
4th	168	95.72	1.76	1.50–2.04
5th	112	61.17	1.83	1.51–2.20
6th	78	44.38	1.76	1.39–2.19
7th	94	35.68	2.63	2.13–3.22

*SMR*, standardized mortality ratio; *CI*, confidence interval

**Table 4:** Cumulative age-specific standardized mortality ratios (SMR) with 95% confidence intervals

Age group (years)	Observed deaths	Expected deaths	Age-specific SMR	95% CI for SMR
40–44	3	0.05	58.40	12.04–170.67
45–49	5	0.42	11.84	3.84–27.63
50–54	12	0.82	14.62	7.55–25.54
55–59	17	2.65	6.42	3.74–10.28
60–64	49	9.11	5.38	3.98–7.11
65–69	76	15.80	4.81	3.79–6.02
70–74	111	28.52	3.89	3.20–4.68
75–79	210	75.96	2.76	2.40–3.16
80–84	333	141.15	2.36	2.11–2.63
≥ 85	644	362.49	1.78	1.65–1.92

*SMR*, standardized mortality ratio; *CI*, confidence interval

SMR estimates in younger age groups, particularly below 70 years, should be interpreted cautiously because of small numbers of observed deaths and low expected mortality in the reference population

In the sensitivity analysis restricted to patients aged ≥ 65 years, cumulative crude mortality increased from 36.3% at 1 year to 44.7% at 2 years, 68.7% at 5 years, and 77.8% at 7 years. The cumulative 7-year SMR was 2.20, while year-specific SMRs remained above 1 throughout follow-up. Data are shown in Supplementary Table 2.

**Fig. 2:**
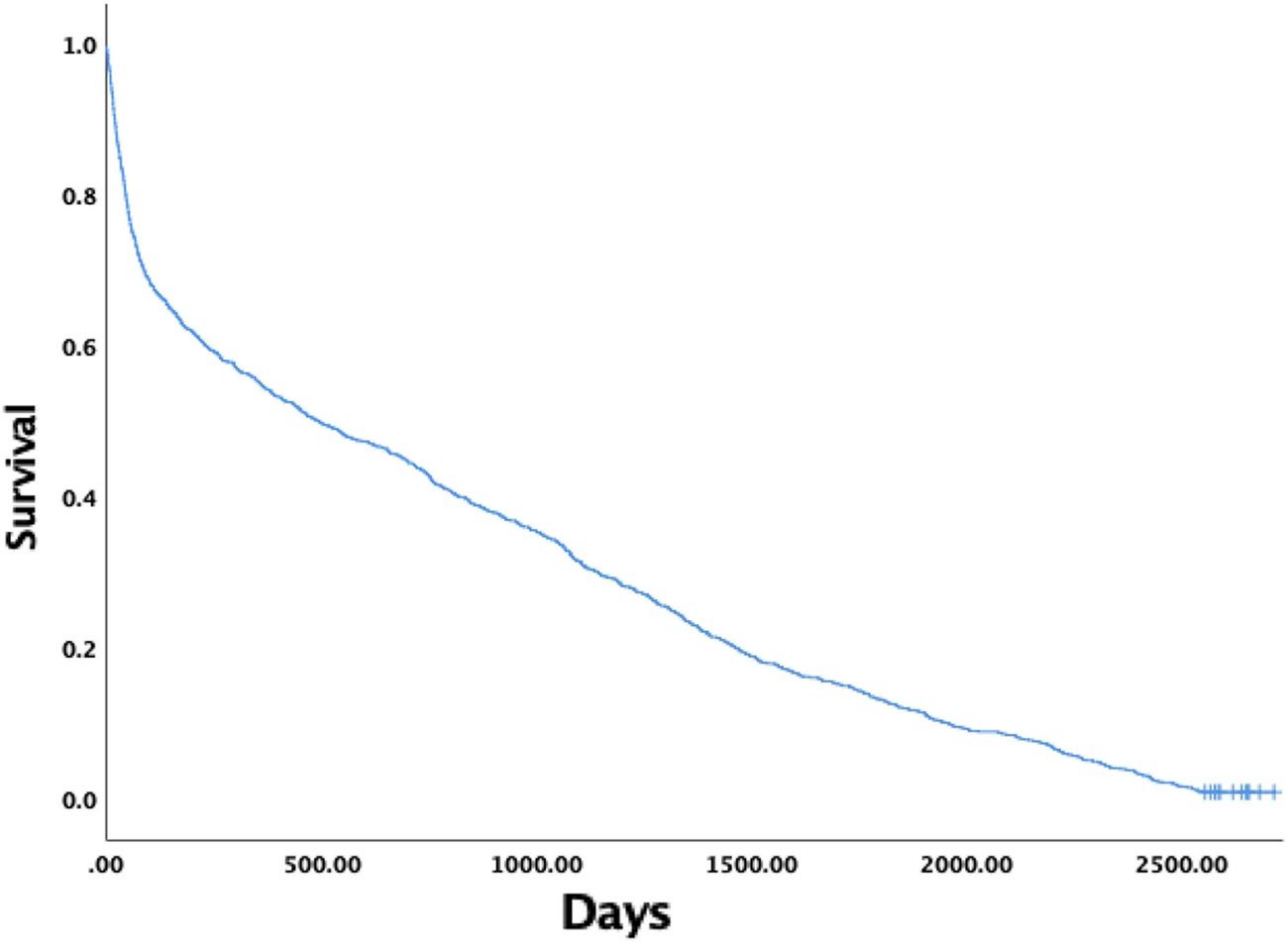
Kaplan–Meier survival curve for the overall cohort of patients with hip fracture. Survival probability is shown over a follow-up period of up to 7 years after the index fracture

Kaplan–Meier survival curves demonstrated a steep early decline immediately following hip fracture, followed by a gradual but persistent decline in survival during long-term follow-up as shown in Fig. 2. Stratified Kaplan–Meier analyses showed that men had consistently lower survival than women across follow-up. Also, fracture type, respectively, extracapsular fractures were associated with lower survival compared with intracapsular fractures and as expected, conservative management was associated with substantially lower survival compared with surgical treatment, with separation of survival estimates occurring early after fracture and persisting thereafter. Kaplan–Meier survival analysis by sex is shown in Supplementary Fig. 1A, by fracture type in Supplementary Fig. 1B and by treatment management in Supplementary Fig. 1C.

Age-stratified Kaplan–Meier curves demonstrated progressively lower survival with advancing age as shown in Supplementary Fig. 2.

## Discussion

The present study builds upon a previously described cohort [5] and quantified the excess mortality after low-trauma hip fracture in the largest urban region in Romania, over a 7-year follow-up. To our knowledge, this is the first study to report long-term excess mortality after low-trauma hip fracture in Romania, by comparing observed mortality with age- and sex-specific population mortality. Apart from our previously published analysis [5] based on the same cohort, which was also the first Romanian study to evaluate post-fracture mortality beyond hospital discharge, no additional data on long-term mortality after hip fracture have been published. The only national-level analysis available in Romania has been limited to in-hospital mortality [11], without longitudinal follow-up or comparison with population mortality rates. Although not nationwide, the present study includes the largest and most heterogeneous region in Romania, encompassing mainly urban but also a smaller adjacent rural area, as well as university teaching hospitals and public hospitals with varying levels of care, thereby providing the most comprehensive assessment of excess mortality after hip fracture currently available in this setting.

We showed that hip fracture was associated with a marked and enduring increase in mortality risk for the entire follow-up period. Approximately 74% of the patients died, and the overall mortality rate in the hip fracture cohort was about 9 times higher than expected based on age- and sex-specific rates in the general population. The excess mortality was most pronounced in the early post-fracture period—the first year after the fracture—but importantly, it did not return to baseline even many years later. Many studies have conformed long-term excess mortality after hip fracture; a population-based data from Singapore demonstrated that excess all-cause mortality can persist up to 5 years post-fracture in both men and women [13].

We found that the standardized mortality ratio (SMR) was extremely high in the initial months after the fracture and, although it attenuated over time, increased throughout the 7-year follow-up period, ranging ~ 1.3–2.0 in years 3–7 depending on age group. This aligns with recent large studies: for example, the CHANCES [2] consortium reported a hazard ratio ~ 2.78 in the first year after hip fracture and still around ~ 1.79 beyond 8 years post-fracture, indicating persistent excess mortality in the long term. Similar persistent excess mortality has been documented in Estonia [14], where hip fracture patients aged ≥ 50 showed significantly higher all-cause mortality over a 10-year follow-up compared with matched controls. Our findings also reinforce the notion that a hip fracture is not just an acute injury but a marker of vulnerability that carries a lasting and persistent impact on survival.

Our observation of sustained excess mortality several years after the index fracture supports the concept that hip fracture identifies a particularly vulnerable patient phenotype. Consistent with this, Tran et al. reported that multimorbidity substantially amplifies excess mortality after fractures, reinforcing that post-fracture mortality is driven not only by the fracture event itself but also by pre-existing health status [8].

To situate our SMR estimates within a health-planning framework, they should be interpreted in the context of the prevailing mortality profile in Romania and Eastern Europe, where cardiovascular disease and stroke remain dominant causes of death. Recent EU statistics indicate that Romania is among the highest-ranking EU countries for mortality attributable to circulatory diseases, while national health system assessments consistently identify ischemic heart disease and stroke as major contributors to overall mortality. Within this broader landscape, the sustained excess mortality observed after post-fracture in our cohort underscores that hip fracture is not solely an orthopedic event, but a condition with substantial population-level impact, supporting the prioritization of integrated orthogeriatric care pathways and robust secondary fracture prevention strategies.

Several patient and fracture characteristics influenced mortality risk, and our results are consistent with patterns reported in the literature. Men in our cohort had higher mortality than women at all time points. This sex disadvantage is well documented in other studies with fewer osteoporotic fractures than women but with greater comorbidity and poorer functional reserve, leading to worse outcomes [1, 11]. While the study of Kannegaard et al. showed that the gender difference in excess mortality in men was particularly significant in the first 3 months after fracture and persisted for up to 36 months after the fracture in patients > 75 years [15], we demonstrated that the difference persists for all the duration of the follow-up in our study (up to 7 years) in all age groups. This difference is currently not fully explained as different hypotheses (the presence in males of higher prevalence of chronic comorbidities, lower overall medication use but increased use of drugs associated with higher risk of fracture) could not account for the excess mortality [15]. A meta-analysis of worldwide cohorts found the mortality risk after hip fracture to be slightly higher in men than in women, although both sexes experience significant excess mortality [10]. Our data support this: the consistently lower survival of male patients suggests that older men with hip fracture should be recognized as an especially high-risk group. They may benefit from more aggressive post-fracture management of medical conditions and targeted rehabilitation, as well as secondary fracture prevention, to improve survival [18, 19].

Advanced age naturally conferred higher absolute mortality in our study, which is expected given age is a strong determinant of death in any population [9]. Although the cohort definition included adults aged 40 years and older for consistency with the parent epidemiological dataset [5, 12], the observed mortality burden was overwhelmingly driven by older adults, which reflects the natural age distribution of low-trauma hip fracture. Therefore, the relevance of the present findings lies primarily in the older population, particularly those aged 65 years and above. However, interestingly, the relative excess mortality is present in also an 85-year-old hip fracture patient, although with a smaller SMR of ~ 1.1–1.3 by the second–fourth year. These results underscore the clinical implication of hip fracture that are present at any age. The long-term mortality patterns observed in our cohort are echoed in the recent Spanish study which included a similar number of patients and found that less than 12% of patients with hip fracture survived to 10 years post-fracture. In both settings, factors such as advanced age and male sex contributed to poorer survival, suggesting common biologic and care-related determinants of long-term outcomes after hip fracture [20]. Compared with the general population, the highest SMR occurred at the first year after hip fracture and then decreased gradually during follow-up, similar to the findings in many other studies [17, 21]. This reinforces the idea that the first year postfracture represents an important widow for active measures to lower the mortality risk.

At the same time, although the actual mortality was higher in the elderly, the excess mortality was higher in younger patients, similar to the results reported by George et al. [16]. This underlines the necessity of close follow-up even in relatively young patients after hip fracture instead of viewing this event as a high mortality event only in the elderly.

Surgical treatment was a critically important modifier of outcome in our cohort. By far, the most striking disparity in outcomes was between patients managed surgically versus non-surgically, difference in mortality that remains evident for the whole follow-up period. The Kaplan–Meier survival curves out to 7 years remained separated by treatment modality, with the non-surgical group experiencing an early and profound mortality toll. Essentially, patients treated without surgery often succumbed in the first weeks and months after fracture and those who did survive the initial period continued to have poorer long-term survival, likely because the very factors that led to nonoperative management (extreme frailty, multimorbidity) continued to limit lifespan. Our findings here are consistent with reports from other settings [22–24]. Countries that have a higher proportion of non-operated fractures, often due to limited resources or delays, tend to report higher mortality rates [25, 26]. An analysis from Estonia, for instance, pointed to their relatively high rate of non-operated hip fractures as a driver of excess mortality, urging improvements in surgical access and decision-making [25]. Our data strongly support prompt surgical treatment of hip fractures in order to increase survival in these patients. This underlines a crucial clinical and public health point for Romania and similar healthcare systems—improving preoperative optimization and expanding surgical capacity to reduce the number of conservatively managed hip fractures could substantially lower early and late mortality. The contrast between operative and nonoperative management underscores the role of care pathways in shaping early mortality and also long-term mortality.

This study addresses an important gap by providing long-term mortality data from Eastern Europe, a region that remains underrepresented in osteoporosis outcome research. While several studies from Central and Eastern Europe, including population-based analyses from Estonia [14, 25, 27] and data from the EuroHOPE project [28], have reported short-term mortality after hip fracture, longitudinal studies are limited. In this context, the present analysis provides novel insight into the long-term survival impact of hip fracture in this region of interest. Also, Romania is currently classified as a moderate fracture risk country based on FRAX estimates [12, 29]; however, the present findings indicate that the mortality burden following hip fracture is substantial. This apparent discrepancy highlights the importance of post-fracture care and secondary prevention strategies, rather than fracture incidence alone, in shaping long-term outcomes. The absence of structured fracture liaison services (FLS) [30] and limited access to coordinated secondary fracture prevention and geriatric rehabilitation may contribute to potentially preventable mortality after hip fracture. 

These results also highlight the importance of strengthening public health strategies aimed at fracture prevention. While improving acute hip fracture care is essential, preventing first fractures through enhanced osteoporosis detection and treatment remains a key priority [31]. In this regard, complementary screening approaches that facilitate the identification of individuals with low bone mass during routine healthcare encounters may improve early diagnosis and broaden access to preventive therapies [32, 33]. Such measures, combined with fall prevention and community-based support, have the potential to reduce the incidence of hip fractures and their associated mortality. Furthermore, the sustained excess mortality observed in our cohort supports the need for prolonged post-fracture surveillance beyond the immediate recovery phase, with structured follow-up enabling timely intervention for recurrent fractures, functional deterioration, and unmanaged comorbidities.

Key strengths of this study include its longitudinal design, complete follow-up of vital status through national registry data, and the inclusion of a large, heterogeneous regional cohort representing real-world hip fracture care in Romania. By linking individual-level fracture data with age- and sex-specific population mortality, this analysis provides the first estimation of long-term excess mortality after hip fracture in this setting, building directly on our earlier work using the same cohort. At the time, this analysis also underscored the lack of an established national infrastructure for longitudinal follow-up of hip fracture outcomes, which currently limits the assessment of long-term mortality at a population level in Romania.

This study has several limitations. First, it is an observational study from a single region. At the same time, Bucharest–Ilfov region is largely an urban area with a particular healthcare infrastructure encompassing an important number of university teaching hospitals; as such, our findings may not fully generalize to rural regions of Romania or other countries. If anything, one might expect outcomes in rural areas (with less access to orthopedic surgery or longer transport times to hospitals) to be even worse, meaning our urban data could represent a “best-case” scenario within Romania. Secondly, we lacked detailed data on patient comorbidities and functional status. This limits our ability to adjust for baseline health differences, especially when comparing subgroups (for example, the non-surgical group was presumably much frailer, and while their mortality is striking, it is partly due to selection bias). Future studies should incorporate comorbidity adjustment to quantify the independent effect of the fracture more precisely. Cause of death data were not available in our dataset (we only had all-cause mortality), which is a common limitation in fracture outcome studies. From a health policy standpoint, all-cause mortality is a relevant outcome since it reflects the real-world burden of hip fracture on survival. The 12-month inclusion period represents another limitation. It was determined by the design of the parent regional cohort and ensured consistency of case ascertainment, inclusion criteria, and baseline data collection. A longer inclusion period would have increased precision, particularly for age-stratified analyses, but would have required a new hospital-by-hospital data collection process and additional administrative approvals, because no centralized national infrastructure currently allows longitudinal post-discharge follow-up of hip fracture patients.

Another limitation of the excess mortality analysis is that reference population data were available only as aggregated tabulated data by calendar year, sex, and 5-year age group, for both resident population counts and number of deaths. Therefore, no individual-level linkage or finer age adjustment was possible. However, mortality rates were calendar year-specific, allowing expected deaths to account for annual changes in background mortality during follow-up. Our study also did not examine subsequent fractures. Suffering a new fracture during follow-up could reset the risk trajectory and contribute to higher mortality [34]. Finally, the study period straddles the COVID-19 pandemic, as noted. We attempted to account for temporal trends in background mortality, but unmeasured pandemic-related factors (like patients avoiding hospitals, or overstretched or limited medical services) might have influenced outcomes in 2020–2021. Flexible parametric survival modelling or Cox models with time-varying effects were not performed. Such analyses could further explore time-varying within-cohort predictors of mortality, particularly during the early post-fracture period. However, because the primary aim of the present study was to quantify excess mortality relative to the general population, the analysis focused on interval-specific SMRs. Also, because mortality after hip fracture changes markedly over time, more flexible modelling approaches allowing time-varying effects (such as flexible parametric survival models) may provide additional insight into temporal risk patterns and should be considered in future analyses.

Despite these limitations, our study provides a necessary foundation for understanding long-term hip fracture outcomes in Eastern Europe. Future research should build on this by expanding to a national cohort, if possible, to confirm that these regional findings hold true country-wide and to identify any regional disparities in outcomes. There is also a need for interventional studies, for example, evaluating whether implementing an organized orthogeriatric co-management service at hospitals in Romania can improve mortality as it was already demonstrated in other countries and also to analyze the excess death that is directly preventable through better fracture care versus underlying health issues that require broader management.

## Conclusions

In summary, this 7-year study confirms that hip fracture in Romanian patients carries a high and lasting mortality burden, with outcomes comparable to the most concerning figures reported internationally. These findings should alert clinicians and health authorities in Romania and maybe also Eastern Europe to strengthen osteoporosis care and hip fracture systems, thereby improving survival and quality of life for this vulnerable and growing patient population.

## Supplementary Information

Below is the link to the electronic supplementary material.

ESM 1 (DOCX 36.0 KB)

ESM 2 (DOCX 14.1 KB)

Supplementary Fig. 1A (PNG 67.6 KB )

Supplementary Fig. 1B (PNG 70.7 KB)

Supplementary Fig. 1C (PNG 75.6 KB)

Supplementary Fig. 2 (PNG 298 KB)
