# Supplementary material for: Excess mortality up to 7 years after low-trauma hip fracture in the largest urban region in Romania
Source: Arch Osteoporos. 2026 Jul 31;21(1):109. doi: 10.1007/s11657-026-01736-3 (PMC13427961; doi:10.1007/s11657-026-01736-3)
Supplement: Supplementary file 1 — (DOCX 36.0 KB) [file 11657_2026_1736_MOESM1_ESM.docx]

# Supplementary Table 1. Age-specific standardized mortality ratios (SMR) by follow-up interval

## First year

| Age group (years) | At risk (start) | Observed deaths | Population rate | Expected deaths | Age-specific SMR |
| --- | --- | --- | --- | --- | --- |
| 40–44 | 6 | 1 | 0.00166 | 0.01 | 100.29 |
| 45–49 | 21 | 1 | 0.00307 | 0.06 | 15.51 |
| 50–54 | 26 | 5 | 0.00652 | 0.17 | 29.52 |
| 55–59 | 46 | 3 | 0.00941 | 0.43 | 6.93 |
| 60–64 | 113 | 19 | 0.01518 | 1.71 | 11.08 |
| 65–69 | 143 | 33 | 0.02324 | 3.32 | 9.93 |
| 70–74 | 177 | 45 | 0.03447 | 6.10 | 7.38 |
| 75–79 | 320 | 80 | 0.05106 | 16.34 | 4.90 |
| 80–84 | 414 | 140 | 0.08903 | 36.86 | 3.80 |
| ≥85 | 711 | 342 | 0.17437 | 123.97 | 2.76 |

## 2^sd^ year

| Age group (years) | At risk (start) | Observed deaths | Population rate | Expected deaths | Age-specific SMR |
| --- | --- | --- | --- | --- | --- |
| 40–44 | 5 | 1 | 0.00163 | 0.01 | 122.70 |
| 45–49 | 20 | 0 | 0.00323 | 0.06 | 0.00 |
| 50–54 | 21 | 0 | 0.00562 | 0.12 | 0.00 |
| 55–59 | 43 | 3 | 0.00995 | 0.43 | 7.01 |
| 60–64 | 94 | 8 | 0.01490 | 1.40 | 5.71 |
| 65–69 | 110 | 13 | 0.02151 | 2.37 | 5.50 |
| 70–74 | 132 | 10 | 0.03191 | 4.21 | 2.37 |
| 75–79 | 240 | 23 | 0.05043 | 12.10 | 1.90 |
| 80–84 | 274 | 32 | 0.08320 | 22.80 | 1.40 |
| ≥85 | 369 | 71 | 0.17435 | 64.33 | 1.10 |

## 3^rd^ year

| Age group (years) | At risk (start) | Observed deaths | Population rate | Expected deaths | Age-specific SMR |
| --- | --- | --- | --- | --- | --- |
| 40–44 | 4 | 0 | 0.00182 | 0.01 | 0.00 |
| 45–49 | 20 | 2 | 0.00386 | 0.08 | 25.88 |
| 50–54 | 21 | 2 | 0.00683 | 0.14 | 13.94 |
| 55–59 | 40 | 1 | 0.01094 | 0.44 | 2.29 |
| 60–64 | 86 | 3 | 0.01698 | 1.46 | 2.05 |
| 65–69 | 97 | 10 | 0.02644 | 2.56 | 3.90 |
| 70–74 | 122 | 11 | 0.04000 | 4.88 | 2.25 |
| 75–79 | 217 | 35 | 0.05798 | 12.58 | 2.78 |
| 80–84 | 242 | 42 | 0.09624 | 23.29 | 1.80 |
| ≥85 | 298 | 72 | 0.19378 | 57.75 | 1.25 |

## 4^th^ year

| Age group (years) | At risk (start) | Observed deaths | Population rate | Expected deaths | Age-specific SMR |
| --- | --- | --- | --- | --- | --- |
| 40–44 | 4 | 0 | 0.00216 | 0.01 | 0.00 |
| 45–49 | 18 | 2 | 0.00390 | 0.07 | 28.47 |
| 50–54 | 19 | 2 | 0.00771 | 0.15 | 13.66 |
| 55–59 | 39 | 7 | 0.01252 | 0.49 | 14.33 |
| 60–64 | 83 | 9 | 0.01956 | 1.62 | 5.54 |
| 65–69 | 87 | 8 | 0.03151 | 2.74 | 2.92 |
| 70–74 | 111 | 18 | 0.04681 | 5.20 | 3.46 |
| 75–79 | 182 | 25 | 0.07256 | 13.21 | 1.89 |
| 80–84 | 200 | 30 | 0.11365 | 22.73 | 1.32 |
| ≥85 | 226 | 67 | 0.21907 | 49.51 | 1.35 |

## 5^th^ year

| Age group (years) | At risk (start) | Observed deaths | Population rate | Expected deaths | Age-specific SMR |
| --- | --- | --- | --- | --- | --- |
| 40–44 | 4 | 1 | 0.00193 | 0.01 | 129.26 |
| 45–49 | 16 | 0 | 0.00333 | 0.05 | 0.00 |
| 50–54 | 17 | 1 | 0.00534 | 0.09 | 11.02 |
| 55–59 | 32 | 0 | 0.01007 | 0.32 | 0.00 |
| 60–64 | 74 | 5 | 0.01469 | 1.09 | 4.60 |
| 65–69 | 79 | 6 | 0.02309 | 1.82 | 3.29 |
| 70–74 | 93 | 15 | 0.03607 | 3.35 | 4.47 |
| 75–79 | 157 | 17 | 0.05421 | 8.51 | 2.00 |
| 80–84 | 170 | 27 | 0.08564 | 14.56 | 1.85 |
| ≥85 | 159 | 40 | 0.19726 | 31.36 | 1.28 |

## 6^th^ year

| Age group (years) | At risk (start) | Observed deaths | Population rate | Expected deaths | Age-specific SMR |
| --- | --- | --- | --- | --- | --- |
| 40–44 | 3 | 0 | 0.00169 | 0.01 | 0.00 |
| 45–49 | 16 | 0 | 0.00299 | 0.05 | 0.00 |
| 50–54 | 16 | 2 | 0.00499 | 0.08 | 25.05 |
| 55–59 | 32 | 2 | 0.00903 | 0.29 | 6.92 |
| 60–64 | 69 | 0 | 0.01322 | 0.91 | 0.00 |
| 65–69 | 73 | 1 | 0.02107 | 1.54 | 0.65 |
| 70–74 | 78 | 6 | 0.03227 | 2.52 | 2.38 |
| 75–79 | 140 | 13 | 0.04925 | 6.90 | 1.89 |
| 80–84 | 143 | 25 | 0.08078 | 11.55 | 2.16 |
| ≥85 | 119 | 29 | 0.17262 | 20.54 | 1.41 |

## 7^th^ year

| Age group (years) | At risk (start) | Observed deaths | Population rate | Expected deaths | Age-specific SMR |
| --- | --- | --- | --- | --- | --- |
| 40–44 | 3 | 0 | 0.00151 | 0.00 | 0.00 |
| 45–49 | 16 | 0 | 0.00277 | 0.04 | 0.00 |
| 50–54 | 14 | 0 | 0.00521 | 0.07 | 0.00 |
| 55–59 | 30 | 1 | 0.00829 | 0.25 | 4.02 |
| 60–64 | 69 | 5 | 0.01314 | 0.91 | 5.52 |
| 65–69 | 72 | 5 | 0.02004 | 1.44 | 3.47 |
| 70–74 | 72 | 6 | 0.03135 | 2.26 | 2.66 |
| 75–79 | 127 | 17 | 0.04975 | 6.32 | 2.69 |
| 80–84 | 118 | 37 | 0.07938 | 9.37 | 3.95 |
| ≥85 | 90 | 23 | 0.16687 | 15.02 | 1.53 |

*SMR estimates in younger age groups, particularly below 70 years, should be interpreted cautiously because of small numbers of observed deaths and low expected mortality in the reference population*
