# Supplementary material for: Excess mortality up to 7 years after low-trauma hip fracture in the largest urban region in Romania
Source: Arch Osteoporos. 2026 Jul 31;21(1):109. doi: 10.1007/s11657-026-01736-3 (PMC13427961; doi:10.1007/s11657-026-01736-3)
Supplement: Supplementary file 2 — (DOCX 14.1 KB) [file 11657_2026_1736_MOESM2_ESM.docx]

**Supplementary Table 2.** Sensitivity analysis of crude mortality and standardized mortality ratios in patients aged ≥65 years

| **Interval** | **Observed deaths** | **Expected deaths** | **SMR** | **95% CI** | **Crude mortality** |
| --- | --- | --- | --- | --- | --- |
| 1st year | 640 | 186.59 | 3.43 | 3.17-3.71 | 36.3% |
| 2nd year | 149 | 105.81 | 1.41 | 1.19-1.65 | 13.2% |
| 3rd year | 170 | 101.06 | 1.68 | 1.44-1.95 | 17.4% |
| 4th year | 148 | 93.39 | 1.58 | 1.34-1.86 | 18.4% |
| 5th year | 105 | 59.60 | 1.76 | 1.44-2.13 | 16.0% |
| 6th year | 74 | 43.05 | 1.72 | 1.35-2.16 | 13.4% |
| 7th year | 88 | 34.41 | 2.56 | 2.05-3.15 | 18.4% |
| **Cumulative 0-7 years** | **1374** | **623.92** | **2.20** | **2.09-2.32** | **77.8%** |
