# Supplementary figures and images for: Excess mortality up to 7 years after low-trauma hip fracture in the largest urban region in Romania
Source: Arch Osteoporos. 2026 Jul 31;21(1):109. doi: 10.1007/s11657-026-01736-3 (PMC13427961; doi:10.1007/s11657-026-01736-3)

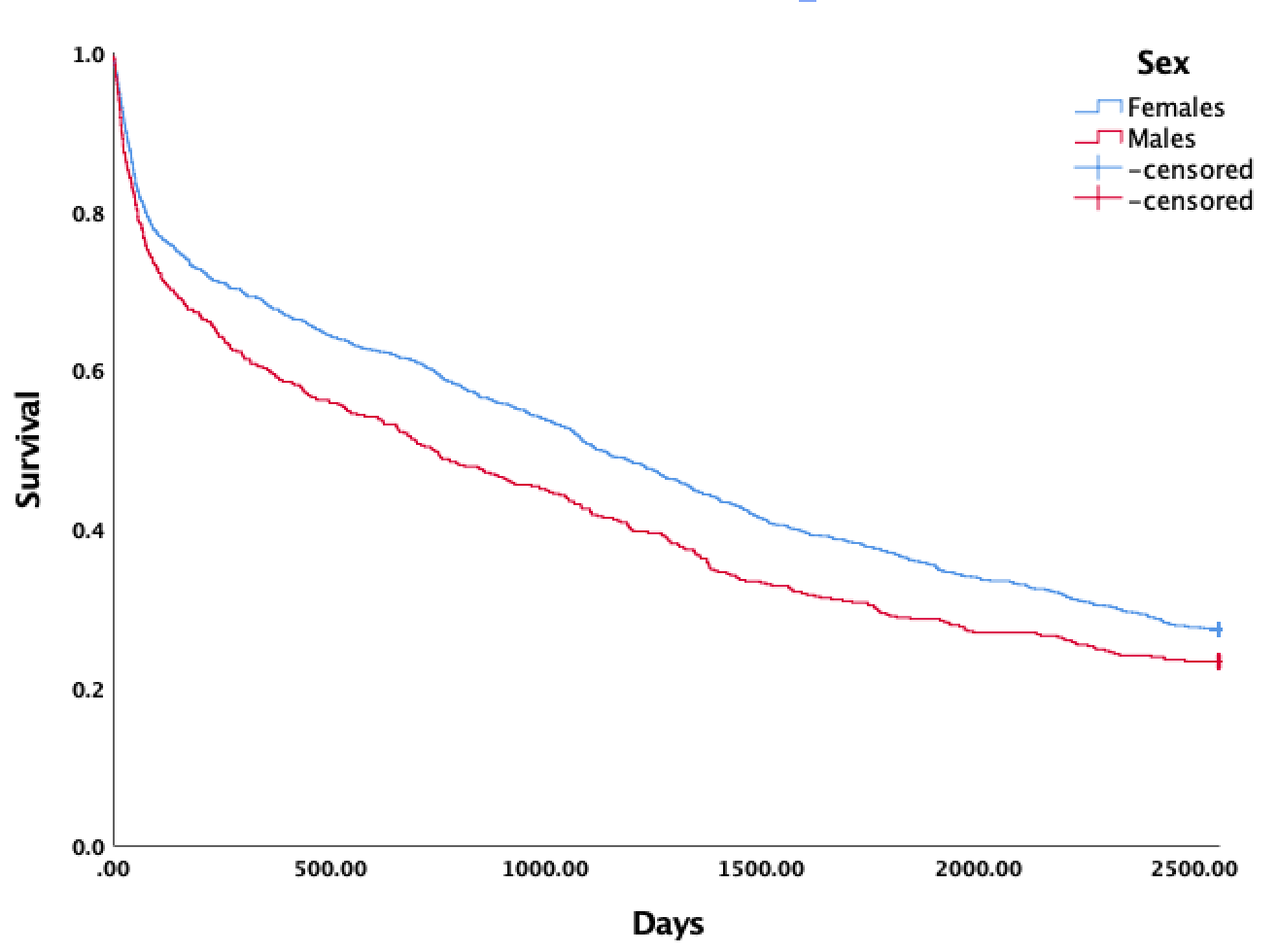

Supplement: Supplementary file 3 — (PNG 67.6 KB ) [file 11657_2026_1736_MOESM3_ESM.png]

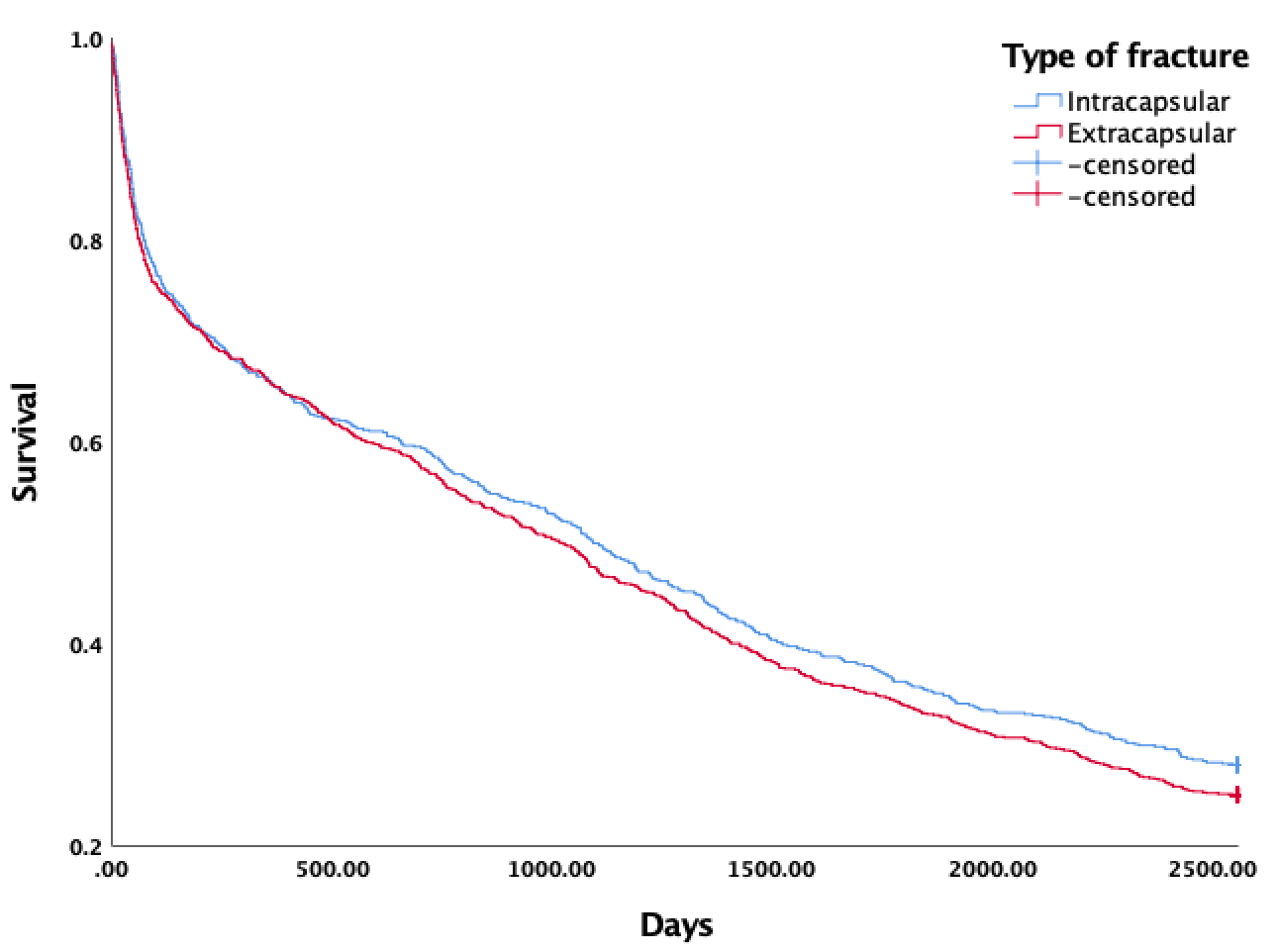

Supplement: Supplementary file 4 — (PNG 70.7 KB) [file 11657_2026_1736_MOESM4_ESM.png]

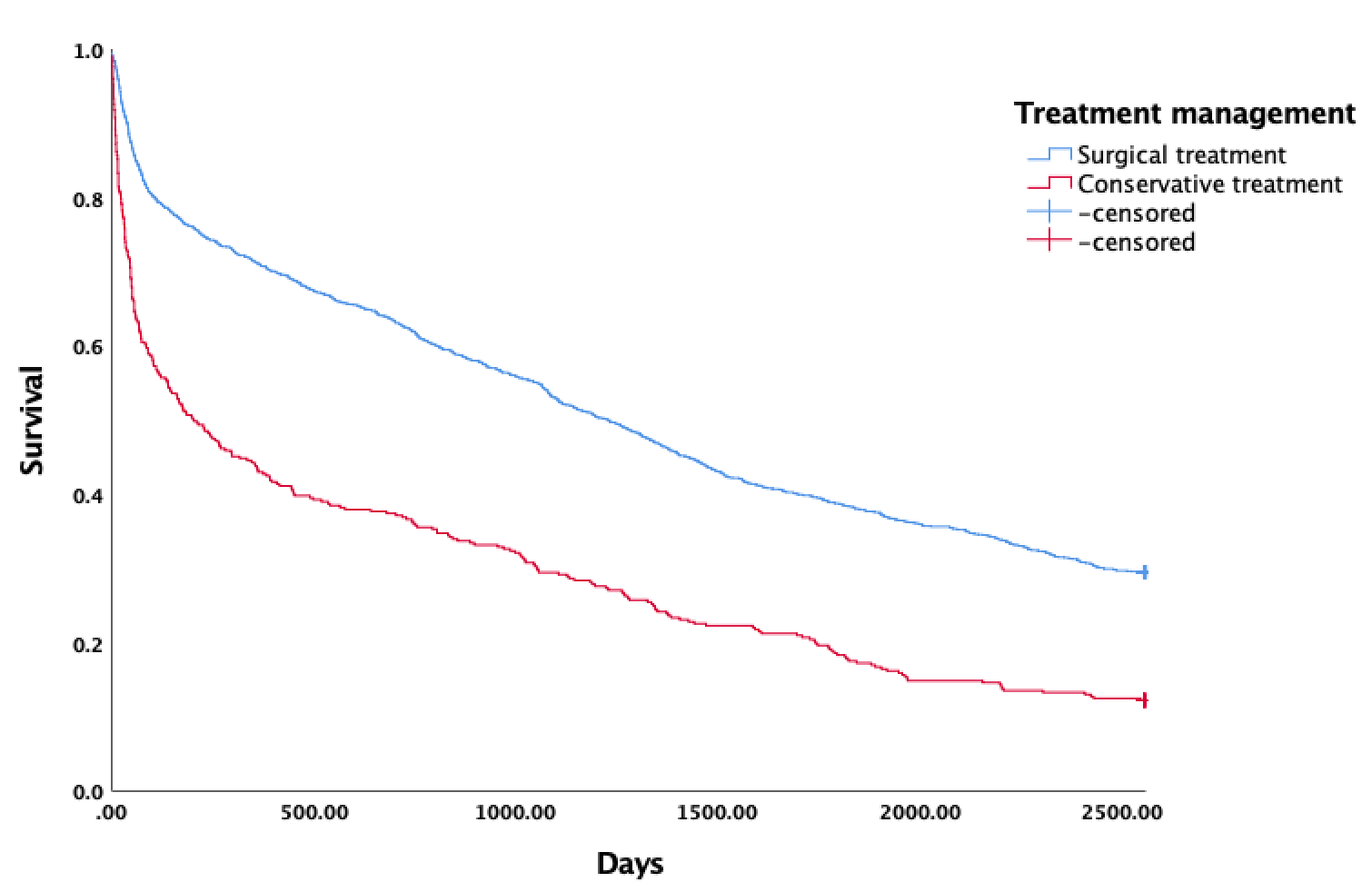

Supplement: Supplementary file 5 — (PNG 75.6 KB) [file 11657_2026_1736_MOESM5_ESM.png]

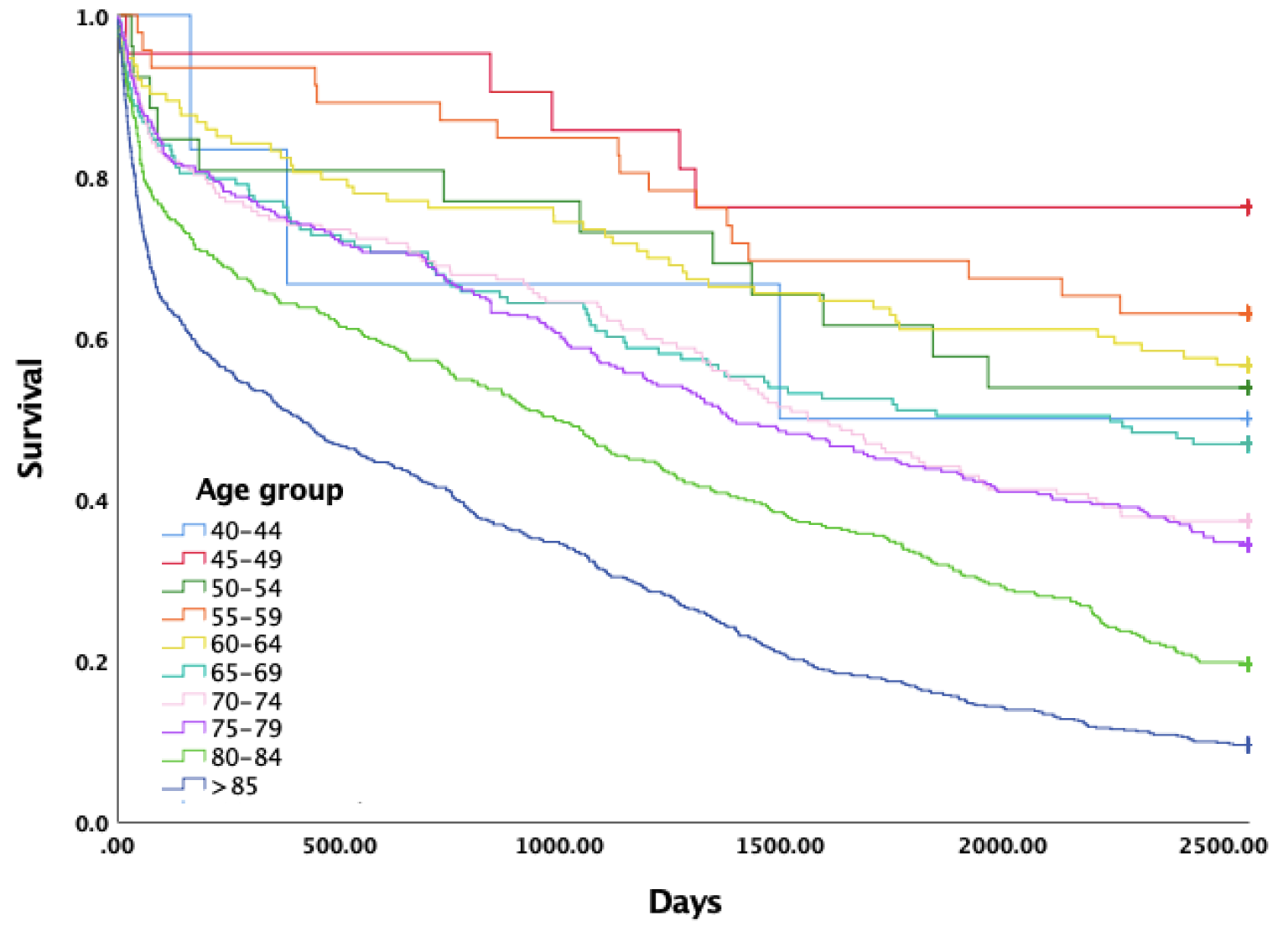

Supplement: Supplementary file 6 — (PNG 298 KB) [file 11657_2026_1736_MOESM6_ESM.png]
